# Bariatric Surgery in Patients with Previous Cardiac Revascularization: Review of Literature

**DOI:** 10.3390/jcm13164779

**Published:** 2024-08-14

**Authors:** Dan Bandea, Ramon Vilallonga, Anamaria Nedelcu, Laura Gabriela Gavril, Marius Nedelcu, Bogdan Andrei Suciu

**Affiliations:** 1 Onesti Municipal Hospital, 601048 Onesti, Romania; dan@bandea.ro; 2 George Emil Palade University of Medicine, Pharmacy, Science and Technology of Tîrgu Mureș, 540142 Tîrgu Mureș, Romania; suciubogdanandrei@yahoo.com; 3 ELSAN, Clinique Bouchard, 13006 Marseille, France; vilallongapuy@gmail.com (R.V.); anamaria.andreica@gmail.com (A.N.); 4 Endocrine, Metabolic and Bariatric Unit, General Surgery Department, Hospital Vall d’Hebron, 08035 Barcelona, Spain; 5 Universitat Autònoma de Barcelona, 08193 Barcelona, Spain; 6 Anesthesia and Intensive Care Unit, Regiional Institute of Oncology, 700115 Iasi, Romania; cotirlet_laura@yahoo.com; 7 Anesthesia and Intensive Care Unit Department, Faculty of Medicine “Gr. T. Popa”, University of Medicine and Pharmacy, 700115 Iasi, Romania

**Keywords:** bariatric surgery, LSG, RYGB, cardiac revascularization, CABG, PCI

## Abstract

**Background**: The diet and physical activity of the world’s population determine the increase in the number of bariatric surgeries. The most common types of bariatric surgery are laparoscopic sleeve gastrectomy (LSG) and Roux-Y gastric bypass (RYGB). Surgical interventions are carried out in patients with numerous comorbidities, among which the most common are cardiovascular diseases. The aim of the present study was to review the literature regarding the safety and results of surgical treatment in patients with cardiac revascularization prior to surgery. **Methods**: We performed an online search in Pubmed in September 2023 to identify articles that reported cardiac revascularization prior to bariatric surgery. The extracted information included details of the working method, number of patients, types of cardiovascular disease—heart failure (HF) and cardiac artery disease (CAD), types of revascularization—coronary artery bypass graft (CABG), percutaneous coronary intervention (PCI) or both CABG + PCI, demographic data (age, gender, race), clinical characteristics (body mass index—BMI, smoking status), comorbidities (diabetes mellitus, hypertension, prior myocardial infarction), postoperative complications, and postoperative results. **Results**: A total of 171 records were identified by the initial search, and 165 papers were excluded after applying the exclusion criteria (types of cardiovascular disease, types of revascularization, and demographic data). We evaluated a group of 9479 patients of which 730 had HF, 2621 CAD, and 1426 underwent prior cardiac revascularization. The analysis of the demographic data showed an average age of 55.5 years and a fluctuation of the male gender between 39% and 71.1%, and the female gender between 28.9% and 61%. The main types of bariatric interventions were RYGB (3659 cases) and LSG (659 cases), to which adjustable gastric band (AGB) and bilio-pancreatic diversion—duodenal switch (BPD-DS) were added. Among the most postprocedural complications were ST-segment elevation myocardial infarction (2 patients), gastro-intestinal bleeding (51 cases), pulmonary embolism (1 patient), arrhythmia (3 patients) and pacemaker insertion (1 patient). The recorded postoperative mortality rate was 0.42% (6 cases). **Conclusions**: Bariatric surgery remains safe in patients with cardiac revascularization. These finding need to be confirmed in more large-scale randomized trials.

## 1. Introduction

In the last three decades, the association between obesity, type 2 diabetes, and cardiovascular disease has been observed more and more often. Calculating the body mass index—BMI shows that over a third of the world’s population is classified as overweight or obese, and it is estimated that by 2025 it will reach 18% of men and 21% of women.

Heart failure (HF) followed the same upward trend. Increased body mass index is an independent risk factor in the development of HF. HF is defined as a clinical syndrome in which morphological and functional changes do not allow the ventricles to fill or evacuate blood. The technological evolution allowed the evaluation of the myocardium based on the ejection fraction (EF) of the left ventricle. Ejection fraction below 40% characterizes systolic HF, and ejection fraction between 40–75% shows diastolic HF. Diastolic HF is common in women and in obesity. Weight loss after bariatric and metabolic surgery (BMS) can lead to improvement of ventricular function observed by echocardiography. These effects occur 3 months postoperatively and reduce major cardiac adverse events (MACE) for more than 10 years. Several obesity studies have shown the “obesity paradox”, in which moderate morbid obesity may be associated with a reduction in mortality and major adverse cardiac events [1].

The ability of adipose tissue to coordinate the storage and release of energy during a meal and between meals is an indicator of metabolic health. In the case of obesity, when there is a positive energy balance for a long period, the possibility of adipose tissue expansion is exceeded, meaning that adipocytes increase in volume, become hypoxic, are infiltrated by inflammatory cells, and reach fibrosis and apoptosis. Thus, the caloric excess can no longer be stored in the usual storage tissues, and the fats are deposited in other tissues (liver, skeletal muscles, pancreas), resulting in lipotoxicity and insulin resistance. Insulin resistance is the main connection between visceral obesity and cardiovascular risk. Obesity increases the risk of heart failure by 11% in men and by 14% in women. Alteration of cardiac function in obese patients is secondary to hemodynamic changes and structural alterations of the heart, along with coronary artery disease, hypertension, dyslipidemia, type 2 diabetes, kidney disease, and obstructive sleep apnea.

Obesity and type 2 diabetes mellitus (T2D) are frequently associated and generate an increased risk of acute cardiac events and HF. In patients with diagnosed heart disease, BS decreases the risk of MACE and mortality. Age, T2D, and HF determine poor long-term weight loss results [2].

BMS through laparoscopic sleeve gastrectomy (LSG), Roux-Y gastric bypass (RYGB) and to a lesser proportion through adjustable gastric banding (AGB), can cause a marked weight loss, as well as remission of T2D, dyslipidemia and hypertension [3]. Regarding the benefit of BMS, there is sufficient data to prove its superiority in treating cardiovascular disease associated with T2D and morbid obesity disease. Despite this, there is limited data regarding the risk of early complications (<30 days) after BMS for this category of patients with cardiac revascularization [3,4,5,6,7,8]. These early complications may fall into two main categories: surgical complications such as leaks and haemorrhages, or cardiovascular events like myocardial infarction and arrhythmias. Consequently, the aim of this study was to evaluate the safety of BS in patients with preoperative cardiac revascularization.

## 2. Materials and Methods

### 2.1. Search Strategy and Study Selection

We performed an online search in PUBMED in September 2023 to identify articles that specify information about patients with cardiac revascularization prior to bariatric surgery interventions. To select the eligible studies, we reviewed the reporting characteristics known for systematic reviews and meta-analyses. We used the following combinations of terms: coronary stent and bariatric surgery, previous cardiac event and bariatric surgery, coronary stent and gastric bypass, and coronary stent and gastric sleeve. Our initial analysis included a prescreen to identify the clearly irrelevant reports by title, abstract, and keywords of the publication. Two other independent reviewers (R.V. and B.A.S.) then assessed the studies for relevance, inclusion, and methodological quality. The studies were classified as relevant (meeting all specified inclusion criteria); possibly relevant (meeting some but not all inclusion criteria); and rejected (not relevant to our review). Two reviewers (M.N. and D.B.) independently reviewed the full-text versions of all studies classified as relevant or possibly relevant. During the initial search in the PUBMED database, we identified 171 articles, of which 165 did not meet the inclusion criteria for the analysis of the title and abstract. The articles were analyzed based on the title, abstract and keywords, and were later divided into three relevant categories: relevant (meeting all the inclusion criteria), partially relevant (meeting some of the inclusion criteria), and irrelevant. The bibliography of all relevant articles was carefully studied to identify information of interest.

We limited our research to articles in English, but there was no limit on the date of publication. Studies of any type that associated bariatric interventions with cardiac revascularization between 2006 and 2021 were taken into account. 

### 2.2. Data Extraction and Management

The extracted data included details of the working method, number of patients, types of cardiovascular disease, types of revascularization, demographic data (age, gender, race), clinical characteristics (BMI, smoking status), comorbidities (diabetes mellitus, hypertension, prior myocardial infarction), postoperative complications, and postoperative results.

### 2.3. Statistical Analyses

We processed the relevant information from the 18 studies remaining after applying the inclusion criteria. Descriptive statistics were used to process the articles, the number of patients, types of surgery, type of cardiovascular disease, type of revascularization, age, sex, and BMI. Efficacy outcomes of interest were synthesized. Because of the high heterogenicity among the studies and the complete lack of randomized controlled trials, a meta-analysis was not deemed appropriate.

## 3. Results

We identified 171 articles that met the criteria for inclusion in the study. The following combinations were used in the search: (coronary stent, bariatric surgery)—18 articles, (previous cardiac event, bariatric surgery)—133 articles, (coronary stent, gastric bypass)—17 articles, (coronary stent, gastric sleeve)—3 articles. After the primary evaluation of the articles found and after analyzing the title, abstract, and keywords, we removed 165 articles, and 6 manuscripts [3,4,5,6,7,8] were considered relevant. In those articles, we revealed the number of patients with cardiac revascularization and the types of revascularization (CABG, PCI, CABG + PCI). From the demographic data, we obtained an average age of 55.5 years and an average BMI of 44,375. The female gender dominated sex statistics. The demographic data for the entire population is summarized in Table 1. 

The six manuscripts selected for a complete analysis in Table 1 included a population of 9479 patients with cardiovascular disease that underwent bariatric surgery. Only in a percentage of 55.4% (5250 patients) the data regarding the cardiac abnormality was found and classified as follows:

- A total of 730 patients (14.04%) were diagnosed with HF
- A total of 2621 patients (49.92%) were diagnosed with CAD
- A total of 1899 patients (36.17%) needed prior cardiac revascularization. The type of cardiac revascularization was accurately identified in only three studies for 1046 patients (Table 2). The percutaneous coronary intervention (PCI) was the dominant type of revascularization with 83.7% (876 cases), the coronary artery bypass graft was used in only 15.5% (162 cases), and the rest of the patients (eight cases) had both types of procedures.

**Figure 1:**
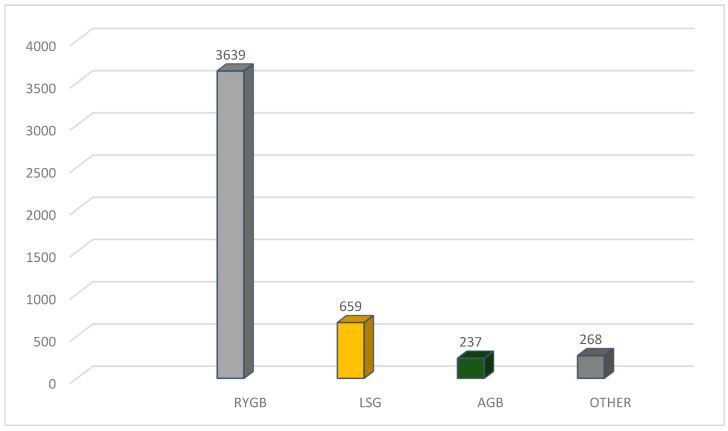
Types of different bariatric procedures.

The type of bariatric surgical interventions (Figure 1) was identified for 4803 cases as follows: 3639 cases with RYGBP; 659 cases of LSG; 237 cases of AGB; and other operations in 268 cases.

Among the most postprocedural complications (summarized in Table 3) were ST-segment elevation myocardial infarction (8 patients), gastro-intestinal bleeding (3 cases), arrhythmia (3 patients), pulmonary embolism (1 patient), and pacemaker insertion (1 patient). The recorded postoperative mortality rate was 0.42% (6 cases). Naslund et al. [3] and Alsabrook et al. [5] each documented a single case of mortality resulting from postoperative bleeding. Doumouras et al. [8] reported an overall 30-day mortality rate of 0.15%, but no further details were provided concerning these four cases. Alsabrook et al. [5] documented one patient who underwent revascularization and another who underwent stenting. The sole case of pulmonary embolism was reported by Okida et al. [4]. Vest et al. [7] mentioned one case of reoperation, while Doumouras et al. [8] identified five cases, yet the cause of reintervention has not been mentioned.

## 4. Discussion

Both the ASMBS (American Society of Metabolic and Bariatric Surgery) and the EASO (European Association for the Study of Obesity) highlight the importance of multidisciplinary evaluation of patients proposed for bariatric surgery. Still, the cardiologist’s evaluation is recommended only for patients with significant cardiovascular history or risk factors, but they do not mandate cardiologist consultation for all patients. Our current practice (France recommendation) includes the cardiologist’s evaluation mandatory for all patients who will undergo bariatric surgery.

The weight loss secondary to bariatric surgery determined the decrease in cardiovascular risk and most importantly in mortality from cardiovascular causes. Following the preoperative and postoperative echocardiographic evaluations, a decrease in left ventricular mass was observed since the first three months after BS. Left ventricular remodeling is independent of hypertension improvement. Postoperative weight loss improved both systolic and diastolic function [1]. Young patients and with advanced HF benefit the most. BS helps patients meet the criteria for registration on the heart transplant list. Therefore, the weight loss associated with bariatric surgery helps to unload the heart both mechanically and metabolically. Seven patients with a left ventricular ejection fraction (LVEF) of ≤25% prior to bariatric surgery were reported by Lim et al. [9]. Six of these patients experienced no major perioperative complications, and their postoperative LVEF improved to a median of 30%. Four patients either successfully underwent cardiac transplantation or met the listing criteria. In three patients, significant improvement in LVEF and functional status was observed, thereby eliminating the need for a transplantation.

Heart failure and prior cardiac revascularization can modify the perioperative outcomes of bariatric surgery. The balance between benefit and risk could be rediscussed for patients with multiple cardiac events. This may also be due to the association of heart failure with lower systolic blood pressure, which is part of the diagnosis criteria of hypovolemic shock. The ongoing review has identified two cases of mortality attributed to haemorrhage following bariatric surgery [3,5]. While we cannot definitively assert an increased risk of bleeding post-bariatric surgery in this patient cohort, it is evident that in instances where haemorrhage occurs, the risk of mortality is more significant within this group.

Different studies [2,3,10] have compared various bariatric surgical procedures. It appears that Roux-en-Y gastric bypass (RYGB) results in more significant weight loss and greater likelihood of diabetes mellitus remission compared to laparoscopic sleeve gastrectomy (LSG). Furthermore, individuals with type 2 diabetes (T2D) who undergo RYGB exhibit lower cardiovascular risk, although the occurrence of long-term postoperative complications is reduced following LSG. 

One of the primary objectives of our present review was to thoroughly examine the data concerning early complications in this patient population, aiming to identify a safer bariatric procedure between RYGB vs. LSG. Unfortunately, the reported complications lack clear identification based on the type of bariatric procedure, rendering it impossible to draw valid conclusions.

Both the overall 30-day complication and reintervention rate vary greatly in the analyzed manuscripts [3,4,5,6,7,8]. In a nationwide cohort study, Näslund et al. [3] investigates the association between metabolic surgery and major adverse cardiovascular outcomes in 509 patients with previous myocardial infarction and severe obesity. They have reported a postoperative complication rate of 8.4%, and 3.8% was classified as serious complications without further details. Doumouras et al. [8] reported an overall 30-day complication rate of 7.7% along with five reoperations. Vest et al. [7] recorded one case of reintervention. However, these studies did not provide specific details regarding the reasons for these reinterventions. Limited information was obtained regarding the number of leaks. Some instances of sepsis could potentially be interpreted as leaks, but no clear data were found for analysis.

In individuals with severe obesity and a history of myocardial infarction (MI), undergoing bariatric metabolic surgery (BMS) not only reduces the risk of major postoperative complications but also improves long-term cardiovascular outcomes—major adverse cardiac events (MACE). When compared to a control group matched for age, sex, BMI, and time since MI, those who underwent bariatric interventions faced less than half the total risk of death, myocardial infarction, or stroke. This suggests that metabolic surgery can serve as a valuable strategy for secondary prevention in individuals with severe obesity [3,4,5,6,7,8,9,10,11]. It seems that not only weight loss alone contributes to reducing the risk of MACE, but so do also all the anatomical and physiological changes secondary to bariatric surgery. A decrease in new onset HF was also observed after bariatric interventions in patients with myocardial infarction [12,13]. Chaudhry et al. [12] reported on six patients with end-stage heart failure who underwent laparoscopic sleeve gastrectomy (LSG). Among these patients, three had previously been fitted with a left ventricular assist device (LVAD). Postoperative complications were observed in one patient, who developed a spontaneous hematoma in the flank region. This same patient also experienced thrombosis in the LVAD pump three weeks after the surgery. This complication necessitated an exchange of the LVAD pump, which was performed without further issues. Remarkably, within 12 months following LSG, all six patients had achieved significant weight loss, making them eligible for heart transplantation. Among them, two patients successfully underwent heart transplantation, while another two were placed on the transplant waiting list. This study highlights the potential of LSG to facilitate transplant eligibility through substantial weight reduction in patients with severe heart failure, even in those reliant on LVADs. Ramani et al. [13] compared 12 patients with systolic heart failure (average left ventricular ejection fraction [LVEF] of 22 ± 7%) who underwent bariatric surgery to 10 matched controls who received only diet and exercise counseling. After one year, the rate of hospital readmissions was significantly lower in the bariatric surgery group compared to the control group (0.4 ± 0.8 readmissions vs. 2.5 ± 2.6 readmissions, *p* = 0.04). Additionally, the surgical group experienced a significant improvement in LVEF, increasing to an average of 35 ± 15% (*p* = 0.005), whereas the control group did not show a significant change, with LVEF remaining at 29 ± 14% (*p* = not significant). Furthermore, within the surgical cohort, one patient successfully underwent heart transplantation, and another was listed for transplantation.

Treating morbid obesity, characterized by a BMI over 40, presents significant challenges, especially when compounded by heart HF. Obesity induces chronic inflammation, leading to endothelial dysfunction and a heightened risk of atherosclerosis. BMS mitigates this inflammation by reducing C-reactive protein levels and elevating plasma concentrations of anti-inflammatory mediators like adiponectin [14,15]. The primary concern regarding BMS lies in its safety for patients with left ventricular systolic dysfunction. In 2016, the International Heart and Lung Transplant Association suggested a BMI below 35 as an inclusion criterion for transplant listing [9,14,16]. Ramanathan et al. [16]. reported on four patients who underwent various bariatric procedures aimed at achieving sufficient weight loss to enhance their eligibility for orthotopic heart transplantation. Remarkably, none of the patients experienced postoperative complications, indicating a high safety profile for these procedures in this high-risk population. In the postoperative period, three out of the four patients successfully attained the necessary weight reduction and subsequently received heart transplants, demonstrating the effectiveness of the bariatric interventions in facilitating access to life-saving transplant surgery. The fourth patient is also listed for transplant upon reaching the requisite weight threshold.

The general mortality rate following bariatric surgery remains relatively low, typically ranging from 0.1% to 0.3% within the first 30 days postoperatively. This rate is influenced by factors such as the specific bariatric procedure performed, the presence of patient comorbidities, and the surgical team’s expertise. Advances in surgical techniques and perioperative care have substantially improved the safety of bariatric surgery, thereby enabling more patients with severe comorbidities, including those with previous cardiac revascularization and heart failure, to undergo these procedures. Our literature review aimed primarily to assess the mortality risk in this specific population. Our findings indicated a mortality rate of 0.42%, which is deemed acceptable given the complexity of these patients’ conditions.

The primary limitation of the present review stems from the incomplete data available in various manuscripts regarding complications arising from bariatric surgery in patients with prior cardiac revascularization, particularly concerning the different procedures of MBS. Regrettably, our review fails to address the crucial question of which bariatric procedure presents the highest safety profile for patients with previous cardiac revascularization. However, our manuscript’s strength lies in its ability to gather more comprehensive data concerning the risks of bleeding and mortality among patients with prior cardiac revascularization. The current data indicates an important risk of mortality in patients experiencing bleeding following different bariatric procedures. Therefore, special attention must be paid to this clinical scenario, emphasizing the need for more intensive management of postoperative bleeding in patients with previous cardiac revascularization.

## 5. Conclusions

Bariatric surgery continues to demonstrate safety in patients with prior cardiac revascularization. However, insufficient data cannot determine which bariatric procedure is safer for this patient group. Our review uncovered a significant risk of mortality associated with postoperative bleeding following bariatric surgery in individuals with prior cardiac revascularization. These findings underscore the need for validation through larger randomized trials.

## Figures and Tables

**Table 1:** Demographic data.

Authors	Year	Patients	Prior CR	Age	Female	BMI
Okida et al. [4].	2020	2884	76	61.4	28.9%	41.7
Alsabrook et al. [5].	2006	77	26	-	-	-
Naslund et al. [3].	2021	509	381	53	42.8%	40.6
Pirlet et al. [6].	2020	116	116	-	-	-
Vest et al. [7].	2016	76	24	51.6	50%	48.2
Doumouras et al. [8].	2021	2638	660	56	61%	47

**Table 2:** Type of prior cardiac revascularization.

Authors	Prior Cardiac Revascularization	CABG + PCI	CABG	PCI
Okida et al. [4]	76	8	30	38
Naslund et al. [3]	738	-	34	704
Pirlet et al. [6]	232	-	98	134

Coronary artery bypass graft (CABG), percutaneous coronary intervention (PCI).

**Table 3:** Postoperative complications.

Postoperative Complications	Total
Minor complications	
ST-segment-elevation myocardial infarction	8 (0.56%)
supraventricular/ventricular arrhythmia	3 (0.21%)
pacemaker/defibrillator insertion	1 (0.07%)
**Major Complications**	
pulmonary embolism	1 (0.07%)
CABG	1 (0.07%)
PCI	4 (0.28%)
GI bleeding	3 (0.21%)
**death**	**6 (0.42%)**

## Data Availability

Not applicable.
